# Estimating Adult Mortality in Papua New Guinea, 2011

**DOI:** 10.1186/s12963-019-0184-x

**Published:** 2019-04-18

**Authors:** Urarang Kitur, Tim Adair, Alan D. Lopez

**Affiliations:** Melbourne School of Population and Global Health, University of Melbourne, Melbourne, Carlton, Victoria Australia

**Keywords:** Papua New Guinea, Adult mortality, Orphanhood method, Socio-economic inequalities, Sub-national mortality

## Abstract

**Background:**

Mortality in Papua New Guinea (PNG) is poorly measured because routine reporting of deaths is incomplete and inaccurate. This study provides the first estimates in the academic literature of adult mortality (45q15) in PNG by province and sex. These results are compared to a Composite Index of provincial socio-economic factors and health access.

**Methods:**

Adult mortality estimates (45q15) by province and sex were derived using the orphanhood method from data reported in the 2000 and 2011 national censuses. Male adult mortality was adjusted based on the estimated incompleteness of mortality reporting. The Composite Index was developed using the mean of education, economic and health access indicators from various data sources.

**Results:**

Adult mortality for PNG in 2011 was estimated as 269 per 1000 for males and 237 for females. It ranged from 197 in Simbu to 356 in Sandaun province among men, and from 164 in Western Highlands to 326 in Gulf province among women. Provinces with a low Composite Index (Sandaun, Gulf, Enga and Southern Highlands) had comparatively high levels of adult mortality for both sexes, while provinces with a higher Composite Index (National Capital District and Manus) reported lower adult mortality.

**Conclusions:**

Adult mortality in PNG remains high compared with other developing countries. Provincial variations in mortality correlate with the Composite Index. Health and development policy in PNG needs to urgently address the main causes of persistent high premature adult mortality, particularly in less developed provinces.

## Introduction

Papua New Guinea (PNG) is a country of eight million people with relatively high mortality and substantial socio-economic and geographic differences [1]. Previous research into mortality in PNG has primarily focused on childhood [2–5] and maternal mortality [6] and in particular infectious diseases such as pneumonia and tuberculosis [7, 8]. Although these have justifiably been topics of considerable and persistent relevance for population health in PNG [9], there has been very little attention paid to the measure of adult mortality, and its causes, despite its importance in overall premature mortality in populations like PNG [10].

The gold standard source for estimating mortality at the national and subnational level is high-quality, complete vital registration data [11]. The lack of empirical data on deaths in PNG to directly measure adult mortality has necessitated the use of demographic and statistical modelling methods that generally use little, if any, local data and instead are driven by model life tables and/or regression modelling with socio-economic covariates [10, 12]. PNG national adult mortality estimates (i.e. the probability of dying between ages 15 and 60 years, or 45q15) for 2011 according to the UN were 266 per 1000 for males and 206 for females,^1^ while Global Burden of Disease Study (GBD) estimates were higher, at 392/1000 for males and 338/1000 for females. Moreover, these mortality estimates are limited to the national level; there is no reliable evidence on adult mortality at the sub-national level despite the fact that significant mortality differentials are highly likely to exist, given the socio-demographic profile of the country.

Given the critical importance of accurate adult mortality measurement to inform population health policy to reduce premature deaths, we set out to estimate adult mortality (45q15) by sex at the national and provincial level in PNG using demographic methods applied to data on parental survival in the 2000 and 2011 population censuses. The second objective is to discuss geographic inequalities and sex differentials in adult mortality risk at the provincial level with reference to a summary index of education, economic and health access indicators to assess the plausibility of our findings.

## Methods

### Data sources

There is no single dataset that accurately describes adult mortality in PNG. Multiple disparate data sources exist, the most important of which are described below. A full summary is shown in Table 1.

**Table 1:** Sources of adult mortality data, PNG

Data Source	Institution	Deaths	Strengths	Weaknesses
Population-Based				
Civil Registration System	Civil and Identity Registry Office (CIRO)	Less than 1% registered annually	Potential source of comprehensive mortality data by age and sex	Dysfunctional system, mortality data held in departmental silos so unable to obtain meaningful and reliable mortality indicators from registered deaths
PNG National Censuses (2000 and 2011)	National Statistics Office (NSO)	Parental survival and summary birth history (SBH) data; 89,000 reported deaths in household in last 12 months (2011)	Data on parental survival to estimate adult mortality (45q15). Summary Birth Histories (SBH) for under 5 mortality (5q0) estimation.	Household deaths have age reporting issues; hence, age-specific death rates are implausible. No father’s survival data in 2000.
Demographic & Health Surveys	National Statistics Office		Potential source of data for adult and child mortality	2006 child survival data is of poor quality. No reliable adult mortality data.
Health and Demographic Surveillance System (HDSS) sites (Partnership in Health Project, Hiri-West, Hides, Asaro and Karkar)	Papua New Guinea Institute of Medical Research (PNGIMR)	1,308 deaths (2009-2014)	Relatively complete recording of deaths within HDSS sites.	Deaths only from specific sites, so not representative of the whole country to enable all-cause mortality analysis
Facility-Based				
National Health Information System (NHIS)	National Department of Health	41,405 deaths (2010–2015)	Deaths recorded from 636 health centerres and 22 hospitals	Age is not recorded in 72% of deaths limiting its usefulness in mortality analysis. Deaths for only those accessing facilities (28%) of all deaths, not sufficient for mortality analysis.
eNHIS	National Department of Health	2487 deaths (2015–2017)	Age recorded for all deaths, timeliness and quality of data	Reported deaths from facilities in only 5 provinces, insufficient for analysis. Facility-based data insufficient for all-cause mortality analysis
Discharge Health Information System (DHIS)	National Department of Health	38,303 deaths (2007–2013)	Records deaths by age and sex from all hospitals and health facilities	Reported deaths account for only 22% of all deaths, so insufficient for mortality analysis.

#### Civil registration and vital statistics system

The civil registration and vital statistics (CRVS) system in PNG is dysfunctional, and birth and death registration remain very incomplete and inaccurate [13]. The system is fragmented as data are kept in departmental silos with little or no coordination and communication between the agencies involved at the central level, including the Civil and Identity Registry Office (CIRO), National Department of Health (NDOH), Department for Provincial and Local Government Affairs, Department of National Planning and Monitoring, and the National Statistics Office (NSO). Given the deficiencies in the CRVS system, it is not possible to obtain statistically meaningful and reliable mortality indicators from registered deaths.

#### Census Data in 2000 and, 2011

The 2000 and 2011 population censuses collected data on the survival status of the mother of each household respondent, and the 2011 census also collected data on the survival status of the father of each household respondent. These are known as maternal and paternal orphanhood questions and require a “yes” or “no” response [14, 15]. The proportion of respondents with their mother still alive or father still alive can be tabulated by sex and 5-year age group of the respondent and then used together with the orphanhood method to estimate adult mortality [16]. The 2011 census also collected data on the number of household deaths in the previous 12 months; however, analysis of these data revealed highly implausible age patterns making them unsuitable for analysis of adult mortality. The 2000 and 2011 censuses collected data on summary birth histories (SBHs); that is, women were asked how many children they had ever given birth to and how many had survived. Such data can be used to estimate child mortality [17, 18]. The 2011 census also asked women how many children they had given birth to in the last 12 months, which can be used to generate a proxy for the mean age at child birth for women. Between the 2011 and 2000 censuses, two new provinces were created: Hela (formerly part of Southern Highlands) and Jiwaka (formerly part of Western Highlands). Due to data for these provinces not being available in both censuses, all analyses are based on provinces as at the 2000 census; Hela as part of Southern Highlands and Jiwaka as part of Western Highlands.

#### Demographic and health surveys in 1996 and, 2006

The Demographic and Health Surveys (DHS) in 1996 and 2006 included questions on the survival of mothers and fathers, as well as SBHs. Although the 1996 and 2006 DHS included the paternal as well as maternal orphanhood question, this data was never tabulated and published in any official survey reports [19]. Moreover, Bauze et al. [2] reported that child mortality data from the 2006 DHS were of poor quality, and so they were not analysed in this study.

#### DHIS, NHIS, and eNHIS

The NDOH collects detailed information on births, deaths and causes of deaths using two paper-based parallel and independent systems comprising of the National Health Information System (NHIS) and Discharge Health Information System (DHIS) [20]. The DHIS is the older of the two systems and reports around 6000-11,000 individual deaths annually primarily from provincial hospitals. The NHIS annually reports a summary of 8000-15,000 deaths annually from mainly health centres, but also from hospitals. The reporting of deaths across the two systems is not independent, with several deaths being included in both systems. Moreover, these data are unsuitable for estimating population-level adult mortality because they only include deaths in facilities; the DHIS only captures an estimated 22% and the NHIS 28% of all deaths in PNG [21]. Furthermore, the NHIS does not report the age at death in 72% of these reported deaths, thus further limiting their value for analysis.

An electronic version of the NHIS was trialled in five provinces between 2014-2017 [22], with deaths recorded via mobile phone and transferred to a central server. However, the coverage of this system is still too low for meaningful analysis.

### Analytical methods

Since it is not possible to directly obtain statistically valid measures of male and female adult mortality using data from the CRVS system or facility-based sources, adult mortality estimates for PNG and its provinces were derived using the orphanhood method applied to data from the 2000 and 2011 censuses. Estimates of adult mortality were made for 2011 because it was the year of the most recent census; other analyses were then based around this reference date.

**Fig. 1:**
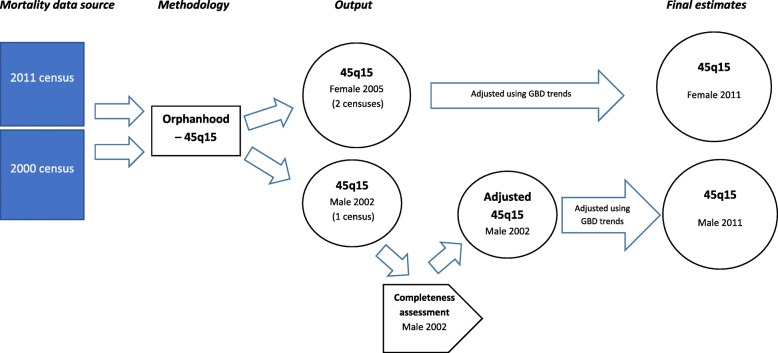
Schematic diagram showing the process undertaken to derive adult mortality

A schematic diagram of the process undertaken to derive adult mortality by province and sex is illustrated in Fig. 1. In summary, 45q15 was estimated for males using orphanhood data from the 2011 census and for females using orphanhood data from the 2000 and 2011 censuses. The completeness of the estimated 45q15 for males was measured using the Adair-Lopez method [23], and this completeness estimate was used to adjust the original 45q15 estimates upwards. The reference date for the male 45q15 was 2002 (an average of the date for each 5-year age group) and for the female 45q15 was 2005; these values were then projected forward to 2011 by applying the trend in PNG 45q15 estimated from the GBD Study[10].

#### Estimating Adult Mortality using the Orphanhood Method

The orphanhood method, first developed by Brass and Hill [24] and refined by Timaeus [25], is a widely used method to indirectly estimate adult mortality. The method estimates male and female 45q15 based on the proportion of respondents with a surviving father or surviving mother, respectively, tabulated by 5-year age group of the respondent. The age of the respondents is used as the duration over which the mother or father has been exposed to the risk of dying and helps to convert the proportion of mothers alive or fathers alive to an estimate of 45q15 [16]. For female adult mortality, the data on the proportion of mothers alive are converted to an estimate of $\frac{l_{25+n}}{l_{25}}$ (where *n* is the age of respondent) using the mean age at childbearing of mothers (calculated from reported births in the last year by age of the mother in the census) and a set of provided coefficients. The relationship of $\frac{l_{25+n}}{l_{25}}$ to *l*_*s*, 25 + *n*_ of a standard population in a chosen model life table^2^ is then used to estimate the female adult mortality rate for each age group of respondents. For male adult mortality, the same process is followed except that the relationship of $\frac{l_{35+n}}{l_{35}}$ to *l*_*s*, 35 + *n*_ is used because men on average tend to be older than their wives. The reference date for the adult mortality for each age group of respondents is calculated based on the mean age of childbearing.

Data on parental survival may be prone to selection bias due to their being based only on reports by surviving children, and if parental and child mortality are strongly correlated then adult mortality would be underestimated [16]. However, such selection bias only arises in populations affected by a generalised HIV epidemic or a severe economic catastrophe, both of which are not the case in PNG [26], and therefore any selection biases are assumed to be minimal. The orphanhood method excludes adults with no living children; however, this is a very low proportion in PNG with only 11% of women aged 40-44 years not having borne any children [15]. Also, if a significant proportion of a provincial population is recent migrants, such as in many subnational populations, then the resultant adult mortality level may also be biased. However, aside from the capital Port Moresby, inter-provincial migration in PNG is relatively low [1] and would not be expected to substantially affect the 45q15 estimates.

The method described above was used to estimate male adult mortality from the 2011 census. Mean age at childbearing of fathers was calculated as mean age at childbearing of mothers plus the difference in median age of currently married males to currently married females [25]. Estimates of male 45q15 and the reference date of the estimates were derived from the average results of respondents aged 10-14 to 30-34 years. Respondents from older age groups were excluded from the calculation because it would have resulted in the reference date being too many years prior to the reference year of 2011.

A variation of the orphanhood method is to use parental survival data by 5-year age group from two censuses and to construct a hypothetical cohort of respondents to estimate adult mortality based on changes in the survival status of each cohort’s parents [27, 28]. This can be done using female mortality in PNG from the 2000 and 2011 censuses. This method provides more accurate estimates of adult mortality than derived from one census alone because it is based on the survival of parents of hypothetical cohorts during an intercensal period; the same information is being asked of the same cohorts of respondents at two points in time, and so any bias introduced by systematic under-reporting of mothers’ mortality would be minimal. The 45q15 is estimated as the average of the 45q15 of each age group 20-24 to 45-49. The reference date for the 45q15 estimates is the geometric mean of the two census dates. The method relies upon the calculation of the mean age of childbearing of the mothers in each census using births in the previous year; these data are only available in the 2011 census and so the mean age at childbearing in 2011 was assumed to apply in 2000 as well.

#### Assessment of completeness of male adult mortality

As mentioned above, there may be under-reporting of male adult mortality derived from applying the orphanhood method to just one census. Under-reporting may occur due to respondents who were adopted not reporting on the survival status of their biological father; adoption of orphaned children by other family members is a common practice in PNG. The completeness of male adult mortality in 2002, the reference date for the male 45q15 estimate, was estimated using a recently developed empirical method [23]. Other widely used methods to estimate the completeness of mortality reporting (death distribution methods and capture-recapture methods) cannot be used in this study because of their data requirements and assumptions of population dynamics [23]. The empirical method requires the following data inputs: the crude death rate based on reported deaths as the numerator, the true under-five mortality rate (5q0; the probability of death from live birth to 5 years) and the proportion of the population aged 65 years and over. The estimated number of reported deaths was calculated using model life tables,^3^ with the 45q15 taken from the orphanhood data and 5q0 for 2002. The 5q0 was obtained by using the 5q0 calculated for each sex and province from the 2000 census from the Maternal Age Cohort method, scaling it to the provincial 5q0 as estimated by Bauze and others, and then scaling these estimates to national 5q0 as estimated by the UN Inter-agency Group for Child Mortality Estimation (IGME) for 2002 [2, 17, 29]. The resultant age-specific death rates were multiplied by the population and summed across all age groups to generate an estimated total number of reported deaths. Population data by age and sex for 2002 were estimated by interpolating between the 2000 and 2011 censuses using a geometric growth rate for the total population and linearly interpolating the age-sex distribution of the population [30]. The 45q15 estimated by the orphanhood method was divided by the estimate of completeness of male adult mortality to produce an adjusted male 45q15 in 2002.

#### Projection of 45q15 estimates to 2011

To adjust both male 2002 and female 2005 45q15 estimates forward to 2011, we used published estimates of 45q15 from the GBD [10]. That is, the ratio of the 2011 to 2002 45q15 as estimated by the GBD was multiplied by the 2002 estimate of male 45q15 to produce 2011 male 45q15, and the ratio of 2011 to 2005 GBD 45q15 was multiplied by the estimate of female 2005 45q15 to produce 2011 female 45q15. The GBD estimates were developed using data and methods independent of the data sources we used in our estimates [10].

#### Data to describe geographic and socio-economic differentials—Composite Index

Variations in mortality may be associated with social, economic and health system characteristics including ethnic group, marital status, education attainment, occupation, income and socio-economic class, as well as access to health services [31]. Disaggregation by socioeconomic indicators is likely to provide a more informative basis for policy action to reduce mortality differentials than simply using rural and urban or provincial classifications [32]. In PNG, which has significant geographic diversity, the use of an index to measure variation in socio-economic and other characteristics is important to interpret and assess differences in mortality. A sociodemographic index (SDI) identified by the GBD [32] has been previously used for comparison of mortality differentials [33, 34]. In PNG, it is possible to rank provinces and districts using a Composite Index derived from socio-economic indicators to interpret differences in mortality at the subnational level. Previous studies [35–40] in PNG have utilised indicators to rank districts and improve targeting of interventions. This study uses a Composite Index calculated as the arithmetic mean of education, economic and health access indicators. The education indicator measures the net admission rate (percentage of children aged 6 years who were admitted to elementary prep school) and female literacy rate [41]; the economic indicator is an average of poverty levels as assessed by the World Bank based on basic food and non-food expenditure [37] and the proportion of people engaged in paid work activities from the 2011 census [15]; the health access index was computed based on information about the number of health workers per population and the immunisation rates from the 2010-2011 Sector Performance Annual Review [42]. The results for each indicator were adjusted to be a normally distributed percentage ranging from 0 to 100%.^4^ We present scatterplots of male and female 45q15 against the Composite Index and show the *r*-squared of these relationships.

## Results

### Adult mortality (45q15)

Table 2 shows the estimated completeness of male 45q15 based on the 2011 census data. Overall, 83% of estimated male deaths in PNG between ages 15-60 were reported in the census, ranging from 62% in Enga to 93% in Central province. The 2011 national male 45q15 was estimated at 269 per 1000, varying from 197 in Simbu province to 356 in Sandaun province. Gulf (346), Southern Highlands (326), Enga (300), Morobe (299), Madang (298), and East Sepik (297) are among the provinces with the highest male 45q15. Conversely, National Capital District (207), Western Highlands (223), Eastern Highlands (228), together with three island provinces (Manus 235, New Ireland 239 and West New Britain 258) reported the lowest male 45q15 for 2011. The estimate of 269 is very similar to the UN’s estimate of 262/1000, but well below the GBD estimate for the same year of 392.

**Table 2:** Male adult mortality (45q15 per 1000) by province, PNG 2011

Province	45q15 2011 is Census (reference year 2002)	Estimated completeness (%)	45q15 Adjusted for completeness (reference year 2002)	45q15 projected to 2011
Western	251	81	291	281
Gulf	322	90	358	346
Central	269	93	293	283
National Capital District	173	81	214	207
Milne Bay	241	87	275	266
Oro	239	85	278	269
Southern Highlands/Hela	229	64	337	326
Enga	206	62	310	300
Western Highlands/Jiwaka	172	71	231	223
Simbu	161	75	204	197
Eastern Highlands	189	79	236	228
Morobe	266	85	309	299
Madang	270	88	308	298
East Sepik	272	85	307	297
Sandaun	336	91	368	356
Manus	215	90	243	235
New Ireland	217	87	247	239
East New Britain	243	85	278	269
West New Britain	227	84	267	258
Bougainville	248	81	292	282
*PNG*	*230*	*83*	*278*	*269*
*GBD*				*392*
*UN*				*262*

For females, the national estimate of 45q15 in 2011 was 237 per 1000, about 12% lower than for males (see Table 3). Provincial estimates vary from 164 in Western Highlands to 326 in Gulf province. Sandaun (305), Morobe (287), Simbu (274) and Enga (272) are estimated to have the highest levels of 45q15 for females. Western Highlands (186), National Capital District (171), Western (181), Bougainville (200), Milne Bay (206) and Manus (211) reported the lowest female 45q15. The national estimate is higher than the UN’s estimate of 202, but again much lower than the GBD’s estimate of 338 [10].

**Table 3:** Female adult mortality (45q15 per 1000) by province, PNG 2011

Province	45q15 2011 and 2000 Census (reference year 2005)	45q15 projected to 2011
Western	191	181
Gulf	344	326
Central	281	266
National Capital District	181	171
Milne Bay	218	206
Oro	233	221
Southern Highlands/Hela	224	212
Enga	287	272
Western Highlands/Jiwaka	196	186
Simbu	289	274
Eastern Highlands	267	253
Morobe	303	287
Madang	273	259
East Sepik	253	240
Sandaun	322	305
Manus	223	211
New Ireland	230	218
East New Britain	262	248
West New Britain	249	236
Bougainville	211	200
*PNG*	*250*	*237*
*UN*		*202*
*GBD*		*338*

**Fig. 2:**
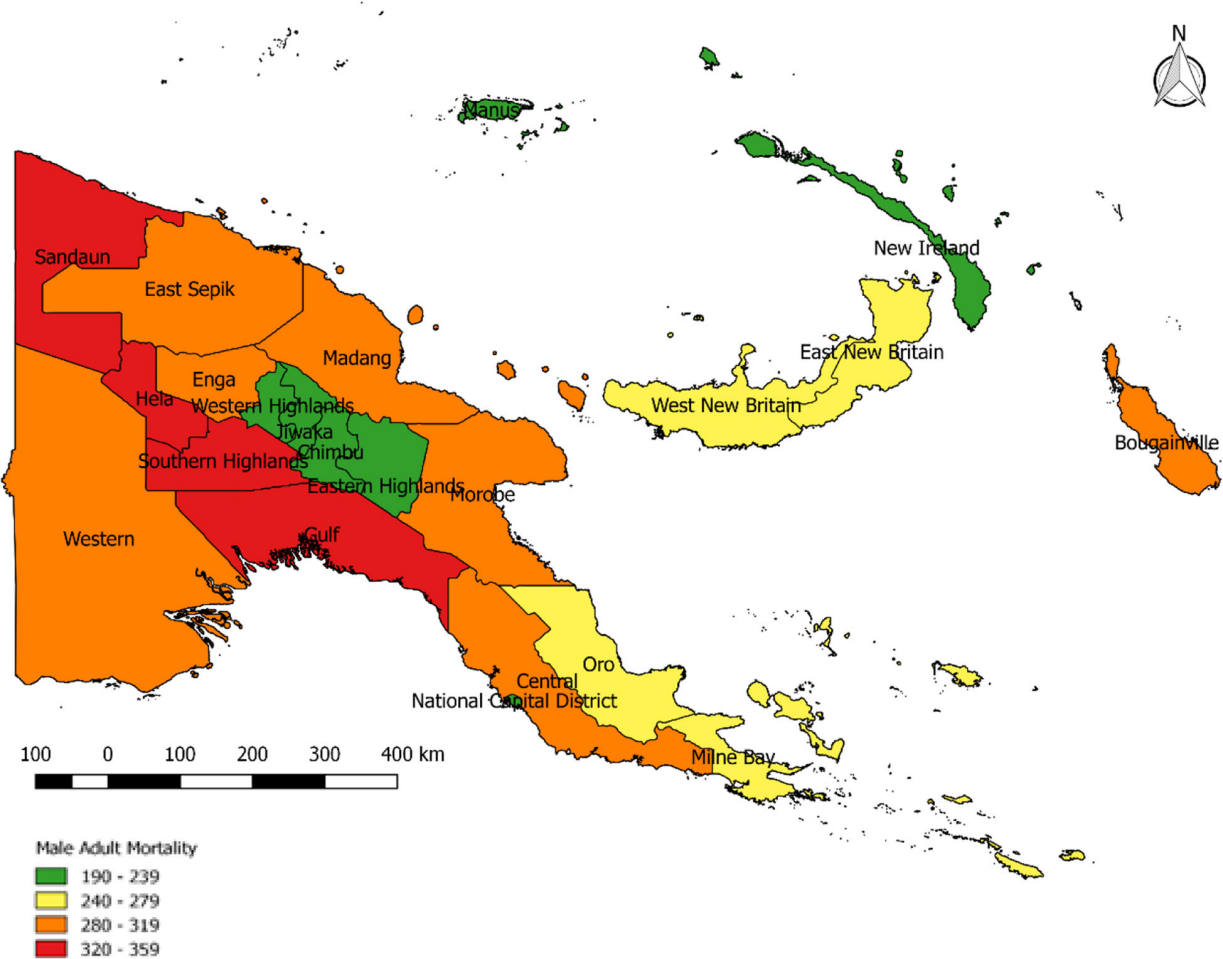
Map of male 45q15 in PNG by province

**Fig. 3:**
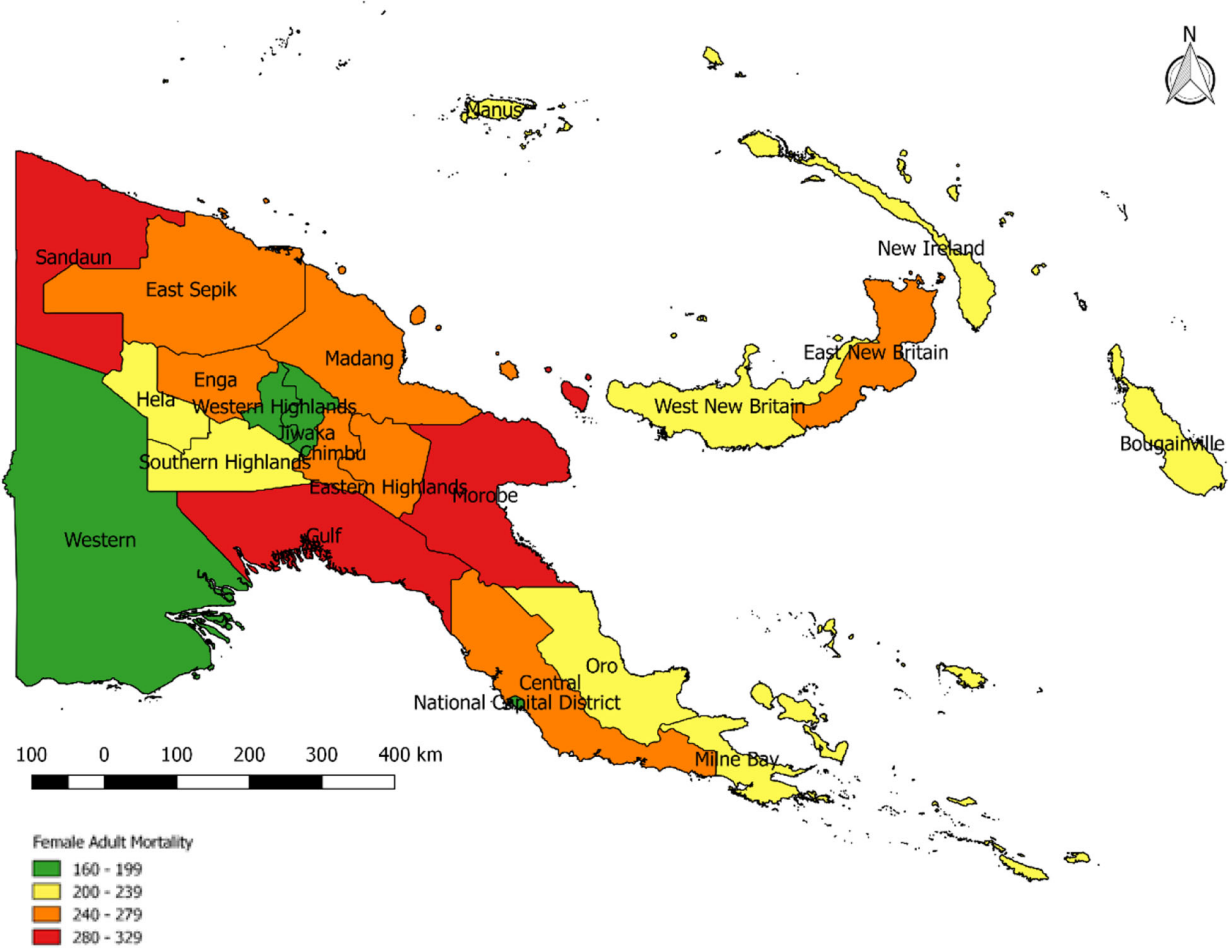
Map of female 45q15 in PNG by province

Figures 2 and 3 show the variation in adult mortality levels by the province for males and females respectively. Among males, there is a belt of comparatively high adult mortality rates running through the central provinces of the country, although interestingly this is not the case in some neigbouring provinces such as Eastern Highlands. The comparatively low levels of adult male mortality prevailing in the eastern coastal (Oro and Milne Bay) and island provinces (Manus, West New Britain, New Ireland) is also clear, with the exception of Bougainville. The northern region (Morobe, Madang, East Sepik) and Bougainville reported moderately high levels of male 45q15. Bougainville was affected by a 10-year-long civil war between 1989 and 1998 which resulted in 15,000-20,000 deaths, mostly men [43]. Central province, despite surrounding the National Capital District, includes Goilala and Rigo, two of the most remote and least developed districts in districts in the country [15, 38].

The national picture for female 45q15 is quite different, however, with much more geographical variation across provinces, although the comparatively low rates in the eastern coastal and island provinces is also apparent for women.

**Fig. 4:**
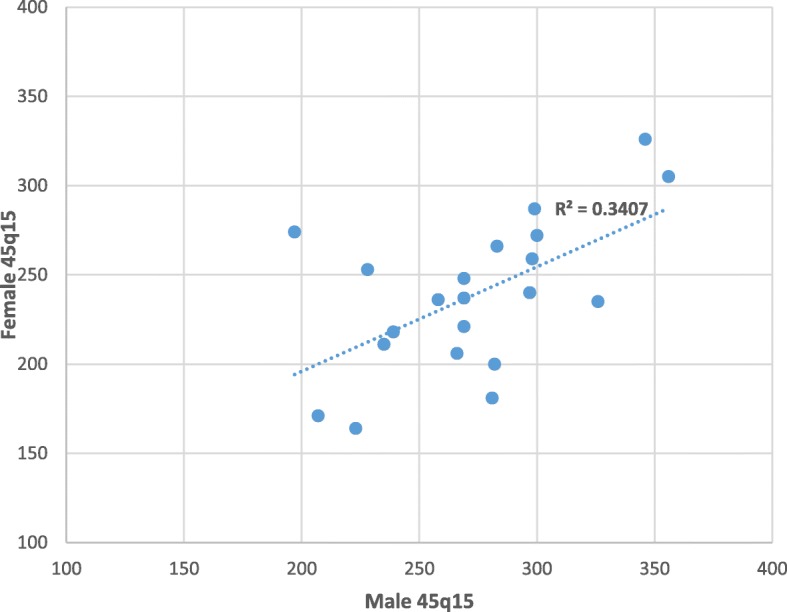
Scatter plot of male 45q15 against female 45q15 in PNG

Sex differences in adult mortality were generally low in PNG as might be expected for a country at an early stage of its NCD epidemic. In general, as female 45q15 increases there is a corresponding rise in male adult mortality (*R*^2^ = 0.3407) (Fig. 4) In all except two provinces, male 45q15 is higher than for females; these provinces are Simbu, where 45q15 is 274 for females against 197 per 1000 for males, and Eastern Highlands where 45q15 is 228 for males and 253 for females. Particularly, large differences in male versus female adult mortality were found in Western, Southern Highlands/Hela and Bougainville. While the civil war on Bougainville could have resulted in high male than female adult mortality, high rates of violence and tribal conflicts in Hela and Southern Highlands could be the reason for the comparatively high excess male mortality compared with females [43, 44].

### Composite Index by province, PNG, 2011

**Fig. 5:**
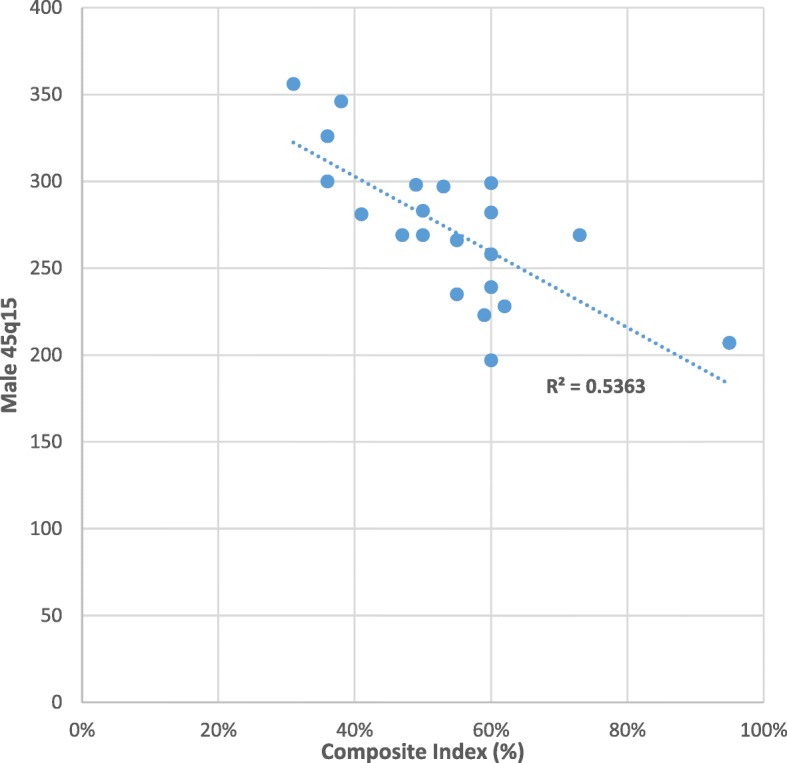
Scatter plot showing the relationship between male 45q15 and Composite Index

**Fig. 6:**
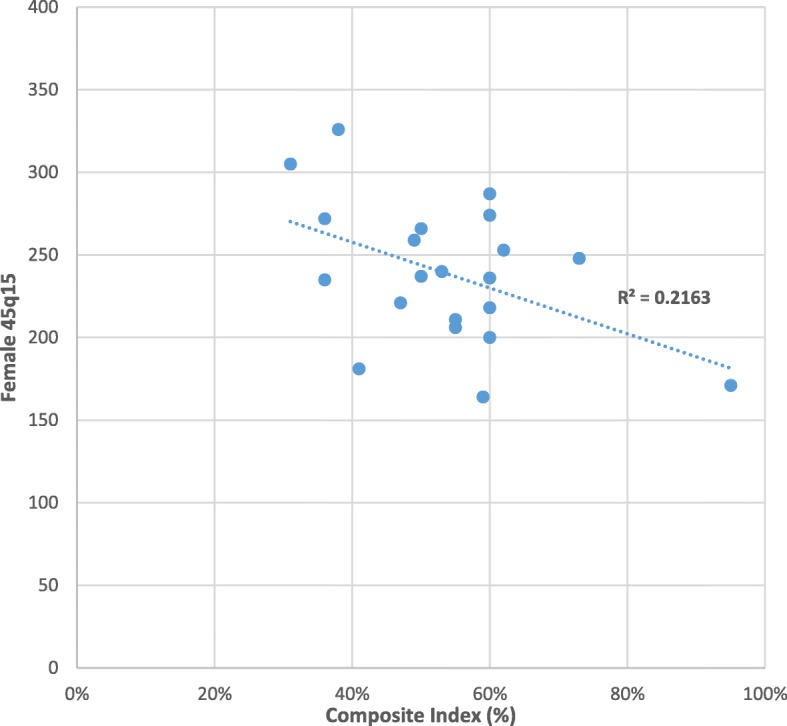
Scatter plot showing the relationship between female 45q15 and Composite Index

Table 4 and Figs. 5 and 6 show the provincial comparison of adult mortality estimates against the Composite Index. In general, provinces with higher composite index (e.g. National Capital District) have correspondingly lower adult mortality, and provinces with lower composite index (e.g. Sandaun, Madang) show higher levels of adult mortality, as expected. The relationship between 45q15 and socioeconomic development is stronger in men (Fig. 5 male *R*^2^ = 0.5363) than women (Fig. 6, female *R*^2^ = 0.2163). For male mortality, there are no clear anomalies; however, for females, the 45q15 for Western is the third lowest while its Composite Index is the fifth lowest.

**Table 4:** Comparison of 45q15 and Composite Index by province, PNG 2011

Province	Male 45q15	Female 45q15	Composite Index (%)
National Capital District	207	171	95
East New Britain	269	248	73
Eastern Highlands	228	253	62
Simbu	197	274	60
Morobe	299	287	60
New Ireland	239	218	60
West New Britain	258	236	60
Bougainville	282	200	60
Western Highlands/Jiwaka	223	164	59
Milne Bay	266	206	55
Manus	235	211	55
East Sepik	297	240	53
Central	283	266	50
Madang	298	259	49
Oro	269	221	47
Western	281	181	41
Gulf	346	326	38
Southern Highlands/Hela	326	235	36
Enga	300	272	36
Sandaun	356	305	31
*PNG*	*269*	*237*	*50*

The lower correlation between 45q15 and socioeconomic status observed for females compared with males could well reflect the fact that men at these ages are more susceptible to the potential negative effects of development than women. These include higher risk factor exposure for non-communicable diseases, less health care seeking behavior, and a reduced risk of violent death [45]. Further research on this male-female variation in mortality responses to development is needed, although interestingly a study by Nikoi and Odimegwu [46] in South Africa found no association between socioeconomic status and adult mortality. Further, our composite index is an aggregate of male and female socioeconomic status and may have concealed important sex differences.

## Discussion

We have estimated adult mortality (45q15) in Papua New Guinea (PNG) by province and sex based primarily on the application of the orphanhood method to 2000 and 2011 census data. To our knowledge, these are the first subnational adult mortality estimates for provinces in PNG derived directly from empirical data. The provincial adult mortality estimates for 2011 show substantial variation, ranging from 197 per 1000 in Simbu to 356 in Sandaun for males and 171 in National Capital District to 326 in Gulf for females. Provinces with the highest 45q15 are found in the Highlands region (Enga and, for males only, Southern Highlands/Hela), Momase (northern) region (Sandaun, East Sepik, Morobe and Madang) and the Gulf in the southern region. These provinces contain some of the most remote communities with very limited access to health services and low socio-economic status; this is reflected by Sandaun, Enga, Southern Highlands/Hela and Gulf having the lowest Composite Index scores. The lowest adult mortality is found in the National Capital District, the island provinces of Manus and New Ireland and the Highlands provinces of Western Highlands/Jiwaka and, for males only, Eastern Highlands. These findings are consistent with the relatively high Composite Index for these provinces, which reflects that they are relatively highly developed provinces with relatively good levels of education and access to services, as well as their low levels of child mortality [2]. More generally, provinces with disparate estimates of adult mortality and child mortality of Bauze et al. are minimal, and there is a stronger relationship between these two indicators for males than there is for females [2].

One province where the findings about 45q15 merit closer inspection is Simbu, which has the lowest male 45q15 (197) and very high female 45q15 (274). While lower male compared with female 45q15 is not implausible, the extent of the difference in Simbu is so large that it is likely due to issues with the relative quality of reporting of female mortality in the 2000 and 2011 censuses or the accuracy of reporting of completeness of male mortality. The Composite Index in Simbu is comparatively high, at 60%, and child mortality is below the national level, which suggests that the relatively low male 45q15 is more plausible than high female 45q15, and so relative quality of reporting of female mortality is more likely the reason [2].

Our estimates of male and female 45q15 in PNG derived from empirical data are broadly consistent with modelled estimates. Existing published estimates of adult mortality levels in PNG are very limited. Our 45q15 estimates for 2011 are 269 for males and 237 for females at the national level; the GBD’s estimates for 2011 were much higher, at 392 for males and 338 for females, based on demographic and statistical modelling that heavily relies on the mortality experience of nearby countries [10]. The UN estimates are 266 and 202 for males and females respectively, based on adult mortality estimated from the parental survival data from the 2000 census, child mortality data from the DHS and the use of model life tables [12]. Our estimates are closer to the UN than GBD, likely because the UN used more local data than the GBD. The high estimates of the GBD may be because it is driven by model life tables based on other Pacific countries with higher mortality due to non-communicable diseases (e.g. diabetes) and it uses modifiers in models to account for the effect of HIV; this could be the reason for the high adult mortality estimates by Rajaratnam and colleagues [47]. We did not include any effect modifiers for HIV or any other diseases since our estimates were based purely on empirical data and because HIV prevalence is relatively low (less than 1% of 15–24-year-olds according to antenatal surveillance) [48]. The National Statistics Office census report estimated 45q15 of 335 for males and 316 for females, based on the use of the hypothetical cohort orphanhood method from the 2006 DHS and 2011 census together with two parameter life tables [19]. However, we did not use the 2006 DHS because of concerns over data quality described by Bauze et al. [2]. Closer assessment of the maternal survival data by cohort in the 2000 census, 2006 DHS and 2011 census showed that the 2006 DHS maternal survival probabilities were much closer to the 2000 census than 2011 census figure, suggesting that estimates for the period 2006–2011 (as used by the National Statistics Office) provide estimates of mortality that are too high.

Our estimates rank male 45q15 in PNG as 142nd out of 195 countries in the GBD and rank female 45q15 as 154th [10]. These are highly consistent with health and development measures for PNG; the UN IGME ranks PNG’s 5q0 as 156th out of 195 countries and the UN’s Human Development Index ranks PNG as PNG 154th out of 188 countries [29, 49]. The ratio of national male to female 45q15 in our study is 1.13; while this is among the lowest (8th) out of 31 countries that the GBD measures as having male 45q15 of between 240 and 300, it is not implausibly low.

In a country lacking an accurate and complete vital registration system, it is not yet possible to measure adult mortality directly [11, 50]. Due to the dysfunctional state of the CRVS [13] in PNG, the civil registration system could not be used for any of the analyses in this paper. This has always been the case with past estimates [2, 19, 51, 52] and will remain so in the foreseeable future given the current status of the CRVS in the country. To use data from the very incomplete and deficient system would portray a very misleading picture of the mortality situation in the country. The NHIS and DHIS, the two most developed death reporting systems in the country, each only report about one-quarter of all deaths in PNG, and these are not representative because they are only facility deaths. Similarly, the 2011 census household deaths yielded low mortality estimates with an implausible age pattern and so were not used for further mortality analysis. It is envisaged that reporting of deaths will improve in the next 2–3 years following the roll out of the eNHIS and with support from the Bloomberg Data for Health Initiative in the country [53].

### Strengths and Limitations

Our estimates have been derived using information from specific questions on parental survival in the 2000 and 2011 censuses. This approach is undesirable in the long term because the census is only held every decade and hence does not provide the timely data that policymakers require. However, at present, there is no viable alternative to estimate provincial-level adult mortality in PNG. The Composite Index shows a moderately strong correlation with mortality estimates. Although it has limited utility to accurately predict adult mortality, it does assist in identifying implausible levels of adult mortality at the provincial level.

A limitation of our methods is that they do not permit the measurement of uncertainty in the mortality estimates. This is likely to be substantial, given the sources of data used, their provenance, and the reliance on GBD estimated age-sex and trend data to project our estimates forward. It is unlikely that the uncertainty around our estimates would be any less than that associated with the GBD estimates, namely ± 20% [10]. It is important that this uncertainty is communicated to users of the data to remind them that while these estimates can provide important policy-relevant insights into comparative levels of mortality, they would be even more useful if significant steps were taken to reduce uncertainty through the establishment of better data systems for the country.

## Conclusion

This study provides, to our knowledge, the first estimates of adult mortality (45q15) at the provincial level in PNG. These show substantial geographic inequalities and sex differentials in adult mortality risk at the provincial level, consistent with provincial differences in socioeconomic development and tribalism. These findings provide policymakers with valuable evidence about levels and patterns of adult population health in a country with limited data and suggest that health and development policy in PNG need to urgently address the large, avoidable causes of premature adult mortality in the country. As a first and critical step in such a policy response, the development of a reliable national mortality surveillance system should be given high priority.

## Abbreviations

CIRO: Civil Identity and Registry Office
CRVS: Civil registration and vital statistics
DHIS: Discharge Health Information System
DHS: Demographic and Health Survey
GBD: Global Burden of Disease Study
NDoH: National Department of Health
NHIS: National Health Information System
NSO: National Statistics Office
PNG: Papua New Guinea
SBH: Summary Birth Histories
SDI: Sociodemographic index
UN: United Nations
UNIGME: United Nations Inter-agency Group for Child Mortality

## Footnotes

1 UN 45q15 figures for 2011 were calculated by interpolating the UN’s 2005–2010 and 2010–2015 figures assuming an arithmetic rate of change.

2 In this study, the UN General model life table is used.

3 The average number of reported household deaths from the UN General, Modified Logit and Log-Quad model life tables was used.

4 Each value was adjusted to a normally distributed percentage ranging from 0 to 100% calculated using the mean and standard deviation of all values in that particular indicator.
